# Using natural experiments to evaluate population health and health system interventions: new framework for producers and users of evidence

**DOI:** 10.1136/bmj-2024-080505

**Published:** 2025-03-28

**Authors:** Peter Craig, Mhairi Campbell, Manuela Deidda, Ruth Dundas, Judith Green, Srinivasa Vittal Katikireddi, Jim Lewsey, David Ogilvie, Frank de Vocht, Martin White

**Affiliations:** 1 MRC/CSO Social and Public Health Sciences Unit, School of Health and Wellbeing, University of Glasgow, Glasgow, UK; 2 School of Health and Wellbeing, University of Glasgow, Glasgow, UK; 3 Wellcome Centre for Cultures and Environments of Health, University of Exeter, Exeter, UK; 4 Health Economics and Health Technology Assessment, School of Health and Wellbeing, University of Glasgow, Glasgow, UK; 5 MRC Epidemiology Unit, University of Cambridge, Cambridge, UK; 6 Population Health Sciences, Bristol Medical School, University of Bristol, Bristol, UK; 7 NIHR Applied Research Collaboration West, Bristol, UK

## Abstract

Natural experiments are widely used to evaluate the impacts on health of changes in policies, infrastructure, and services. The UK Medical Research Council (MRC) and National Institute for Health and Care Research (NIHR) have published a new framework for conducting and using evidence from natural experimental evaluations. The framework defines key concepts and describes recent advances in designing and planning evaluations of natural experiments, including the relevance of a systems perspective, mixed methods, and stakeholder involvement. It provides an overview of the strengths, weaknesses, applicability, and limitations of the range of methods now available, and makes good practice recommendations for researchers, funders, publishers, and users of evidence.

Unlike true experiments that are conducted by researchers for scientific purposes, natural experiments occur when infrastructure, policies, or services are introduced or changed by governments or healthcare systems. Interventions of this kind are sometimes amenable to randomised controlled trials, for example, if the advantages of randomisation can be negotiated with policy makers or providers at the planning stage and the findings are likely to be transferable across several contexts. Although the randomised controlled trial remains an important method, there are occasions when a trial will not be appropriate or feasible for answering questions about infrastructure, policy, or service changes. However, provided that the intervention divides a population into groups that are otherwise similar, researchers can evaluate the health effects of the changes in a natural experimental evaluation. Natural experiments therefore generate valuable opportunities for evaluating population health, health systems, and other interventions, including those that are, for practical or ethical reasons, not suitable for investigation using randomised controlled trials.

One example of a natural experiment is the introduction by the Scottish government in 2018 of a minimum price at which a unit of alcohol could legally be sold. This was expected to reduce alcohol consumption, with most impact on the heaviest drinkers who tend to drink the lowest priced alcohol. A natural experimental evaluation has been conducted comparing trends in alcohol related deaths and hospital admissions in Scotland, before and after minimum unit pricing was introduced, with trends in England, which did not have a similar policy.^1^ In addition to policy changes, natural experimental approaches can be used to evaluate changes to health systems and broader infrastructure. An example of changes to health systems is the study by Doyle and colleagues, which analyses the effectiveness of emergency hospital care using ambulance callouts as a form of quasi-random assignment of patients to different hospitals.^2^ An example of changes to broader infrastructure is the study by Ogilvie and colleagues of the health impacts of a new urban motorway. This study uses a combination of repeat cross sectional and cohort analyses of surveys of residents in intervention and control areas, ethnography, and controlled interrupted time series analysis of routine police road traffic casualty data^3^ (table 1 and table 2 give overviews of the roles of different quantitative and qualitative methods).

**Table 1:** Quantitative methods for evaluating natural experiments

Study design	Level of data collection	Data	Statistical approaches	Overview	Illustrative example* and outcome measure†
Cross sectional	Individual level	Postintervention; random sample (ideally); single time point of data collection; data potentially collected in control group(s)	Descriptive statistics for effect size—with representation of uncertainty; possible matching of intervention group(s) with control group(s)	Allows for estimation of effect, assuming it is “known” what outcomes were preintervention or outcomes are same in intervention and control groups preintervention	Study conducted in all, or subset of, primary care centres postintervention; rate of incident CVD events compared with control group(s) (or compared with literature); possible matching of control group(s) to intervention group(s) before comparison
Repeated cross sectional	Individual level	Preintervention and postintervention; random samples (ideally); data collection at unequally spaced time intervals; no data from control group(s)	Difference between preintervention and postintervention in means, proportions, or rates (depending on nature of outcome measure variable)—with representation of uncertainty; regression models or propensity scores to adjust for confounding variables and/or assess effect modification	Allows for comparison with preintervention outcome, but because pre and post groups include different people this might bias comparisons	Study conducted in all, or subset of, primary care centres preintervention and postintervention (two time points); difference in rate of incident CVD events compared preintervention and postintervention
Before and after	Individual level	Preintervention and postintervention; random sample (ideally); two time points of data collection (on same people—repeat measurements); no data from control group(s)	Average difference between preintervention and postintervention measurements of the outcome measure; regression models or propensity scores—to adjust for confounding variables and/or assess effect modification	Allows for comparison with preintervention outcome based on repeated measures of the same group, but does not have a control group	Not possible given nature of outcome measure (incident CVD events); would be possible if, eg, SBP was outcome measure
Regression discontinuity	Individual level	Random samples (ideally); data collected either side of a “cutoff” for a variable determines if a person is eligible for intervention (and assignment to intervention or control group). An “instrument” is a variable that is associated with exposure to the intervention but not itself associated with outcome	Non-parametric methods; regression models; assess effect modification	Can help minimise bias owing to unmeasured confounding. Limited situations where a cutoff can be identified. Strong instruments are difficult to identify	Use one of the eligibility criteria of health check (SBP>140 mm Hg) as “cutoff”. Use distance to primary care centre where health check is being offered as “instrument”
Difference in differences	Individual or aggregate level	Random samples (ideally); preintervention and postintervention; intervention and control group	Regression models; possible matching of intervention group with control group	Before-and-after design with a control group. Can be difficult to identify comparable control unit(s)	Difference between intervention and control groups in difference in rate of incident CVD events compared preintervention and postintervention
Interrupted time series	Aggregate level	Preintervention and postintervention; data collection on multiple occasions, generally at equally spaced time intervals; “interruption” is at time point when intervention starts; no data from control group(s)	Time series; (s)ARIMA or (panel) regression models; adjustment for confounding variables; assess effect modification	Allows for comparison with preintervention outcome based on several repeated measures of the same group, but does not have a control group	Study time series of rates of incident CVD events; single time series (data from primary care centres combined) or multiple time series (for each, or subgroups, of primary care centres)
Controlled interrupted time series	Aggregate level	Preintervention and postintervention; multiple time points of data collection (generally at evenly spaced intervals); “interruption” is at time point when intervention starts; intervention and control group(s)	Time series; ARIMA or (panel) regression models; adjustment for confounding variables; assess effect modification. Use the preintervention data to create a “synthetic control”; a weighting procedure is applied using the outcome variable and possible confounding variables from pool of control groups	Interrupted time series with control group. Preintervention time period differences between intervention and control groups might cast doubt on intervention effect estimates. If appropriate controls cannot be identified, synthetic control can be developed to obtain counterfactual. Quality of synthetic control not always easy to establish	Study time series of rates of incident CVD events in intervention and control group(s) or synthetic control group

CVD=cardiovascular disease; SBP=systolic blood pressure; (s)ARIMA=(seasonal) autoregressive integrated moving average.

* CVD “health check” delivered in primary care centres (ie, screening for CVD risk factors).

† Incident CVD events (hospital admissions or deaths).

**Table 2:** Contributions of qualitative methods to natural experimental evaluations with examples

Evaluation component	Role of qualitative methods	Data generation or analysis methods used
**Characterising the intervention, context, and system**		
Describing the intervention	Characterised Daily Mile intervention, which encourages children to run for 15 min each school day, by comparing the intervention as described in principle in promotional material with how it was implemented in practice^4^	Ethnographic observation, document review, interviews
	Within a process evaluation, identified important factors that enhanced and hindered implementation and normalisation of a complex intervention in maternity services to reduce smoking rates in pregnancy^5^	Observations, semi-structured and group interviews, analysed using normalisation process theory
Describing the system or context	Used system mapping to describe a complex adaptive system in which a proposed sugar-sweetened beverage levy in the UK would be introduced, and identified key stakeholders’ perspectives on its likely impacts^6^	Expert workshop, Delphi exercise, and qualitative interviews
Developing theories of change	Informed logic model for evaluation of proposed Graduated Driving Licensing in Northern Ireland using focus group analysis to inform choice of plausible comparator settings, hypothesise links between intervention and potential public health impacts, and identify adoption of telematics insurance products as a co-occurring intervention in system^7^	Group interviews analysed by thematic content analysis
Informing selection of populations, controls, and subgroups for analysis	Informed appropriate dates for measuring change resulting from intervention, and identified important subgroups for analysis of effects in evaluation of Cambridgeshire Guided Busway (new bus network, traffic-free walking, and cycling route), by identifying that intervention was used before being officially open, and that previous experience of different modes influenced initial perceptions^8^	Interviews, media analysis, photo elicitation, and participant observation
	Used local practitioners’ insights on appropriate comparator areas to contribute to creating synthetic controls as counterfactuals for evaluation of impact of alcohol licensing decisions on local health and crime^9^	Consultation with local practitioners
Characterising and selecting outcomes and indicators	Refined outcome indicators in evaluation of free bus travel by identifying that travelling by bus entails considerable physical activity for young people, so “bus trips” as an indicator of passive travel would underestimate “active travel”^10^	Ethnographic observation
	Refined interpretation of outcomes measured in survey questionnaire of commuters through interview data, which suggested that some survey respondents’ negative reports of walking and cycling environments reflected desire to make political point about poor facilities, rather than necessarily representing their own perceptions^11^	Photo-elicitation; content analysis of interviews after questionnaire
Generating data on outcomes	Gathered evidence of outcomes not identifiable through routine datasets for evaluation of impact of reduced street lighting at night, such as experiential and behavioural impacts on wellbeing of light and dark at night^12^	Individual and group interviews, media analysis
	Identified important and unanticipated outcomes of dance mats to increase physical activity in secondary schools, including improved reaction times and coordination skills, and acceptability to older girls^13^	Before and after interviews and focus groups
Understanding mechanisms and mediators	Identified possible reasons for lack of effect on health behaviours or physical activity in older adults of low cost improvements to local urban green spaces in Manchester, UK: that small green spaces were seen as belonging to others, and that residents preferred larger parks^14^ ^15^	Walk along interviews and photo-elicitation
	Developed understanding of limited efficacy of smoke-free schools policies in reducing adolescent smoking through analysis of discourses of school children in seven European countries, which identified that policies were associated solely with “the school,” and that they displaced smoking to other spaces^16^	Critical discourse analysis of focus group data
	Explained impact of health system context on outcomes in evaluation of mergers of urology departments in Denmark, in which readmission rates went down, but length of stay increased after restructuring; qualitative analysis suggested that expected efficiency gains of centralisation are undermined in contexts of cost constraint and external pressure^17^	Interviews with service providers and institutional theory
Explaining change	Tested plausibility of “signalling effect” (ie, policy debate itself draws attention to an issue among public and triggers behaviour change) as a mechanism through which taxes on sugar-sweetened drinks in Barbados reduced sales^18^	Process tracing, using television news archives, interviews, the public, and point of sale data
	Identified mechanisms of change and necessary components for transferability of intervention to provide free bus travel, through comparisons within qualitative data (such as deviant cases who reported lack of access to a pass or lack of ability to use buses easily)^19^	Inductive qualitative analysis of focus group, interview, and observational data
	Explained subgroup differences in outcomes of social prescribing intervention for people with diabetes by analysing how “health capital” and structural conditions shaped participants’ capacity to interact with and benefit from intervention^20^	Ethnography involving interviews, photo-elicitation, and participant observation
Understanding stakeholders’ perspectives	Identified unexpected perceptions of residents in evaluation of impact of 2012 Olympics in London, who felt safer with, rather than marginalised by, enhanced security in their neighbourhoods^21^	Family narrative interviews, go-along interviews, focus group workshops
	Produced evidence to explore unexpected effects of covid-19 pandemic on implementation of mass transit cable car in Bogotá, Colombia by exploring residents’ and policy stakeholders’ perspectives on likely impacts and historical context of intervention^22^	Citizen science methods, involving public volunteers in planning, conducting and analysing evidence
	Identified limitations in likely effectiveness of future transferability of home energy efficiency interventions in England by analysing householders’ motivations for installation, which identified that current policy framings around “environmental sustainability” resonated poorly^23^	Household interviews

Guidance on using natural experiments was published by the UK Medical Research Council (MRC) in 2012,^24^ and several other overviews of methods and approaches have been published since then.^25^ ^26^ ^27^ In recent years, there has been a substantial increase in the number of evaluations of natural experiments, advances in analysis methods, and in the application of whole system approaches to evaluation, greater availability of large administrative or routinely collected datasets, and demand for evaluation of natural experiments delivered at a population level. Whereas the 2012 guidance and subsequent overviews have focused on quantitative methods for measuring the effect of interventions, we believe there is a need for a broader framework that also considers the design and planning of natural experimental evaluations, the role of qualitative, mixed methods and economic evaluation, the use of routinely collected data, and the implications for evidence synthesis.

In this article, we present a new framework that provides an integrated guide for using a natural experimental approach to evaluating population health and health system interventions, covering the whole process from study design and planning through to reporting and dissemination. The framework provides a resource for researchers conducting evaluations, users of evaluation evidence, and evaluation commissioners deciding whether a natural experimental approach would meet their needs. The framework also provides information to help journal editors, funders, and peer reviewers understand the strengths and limitations of funding proposals and articles reporting natural experimental evaluations. A detailed version of the framework funded by the UK MRC and the National Institute for Health and Care Research (NIHR) has been published by the NIHR Journals Library.^28^

**Summary points**

Natural experiments that occur when policies, services, or infrastructure are introduced, modified, or withdrawn generate valuable opportunities for evaluation
The UK Medical Research Council and National Institute for Health and Care Research published a new framework to help producers and users of evidence make the best use of natural experiments
The new framework updates and extends previous guidance by taking account of recent developments in approaches to evaluating complex interventions and showing how they can be applied in natural experimental studies
Recommendations for good practice include the use of mixed methods, careful attention to the context in which interventions take place, stakeholder engagement, open science practices, and continued investment in research data infrastructures

## Development of the framework

We convened a writing group of researchers with expertise in epidemiology, health economics, public health and sociology, and methodological expertise in statistics, qualitative research, and evidence synthesis. An advisory group of stakeholders experienced in using natural experimental evidence and with methodological expertise provided input at key stages of the project. We held online workshops and a consultation to obtain expert opinion on developing the framework. The workshops helped configure initial drafts of the framework, then during the consultation, participants were invited to review each chapter of the framework, with the additional feedback used to further refine the content. Participants in the workshops (n=21) and consultation (n=44) comprised researchers and other relevant stakeholders in Europe, Africa, the Americas, and Australasia, including members of research funding boards, policy makers, journal editors, and representatives of national and local governments. Further details of the methods used to develop the framework are available in online supplementary file 1 and the detailed report.^28^ The workshops and consultation helped guide the use of a broad definition of natural experiment, refine a framework for planning and conducting evaluations of natural experimental studies, and specify in detail the role and content of analytic methods in evaluations.

## Framework for natural experimental evaluations

Natural experimental evaluations are valuable for many reasons. They can be used to study interventions under real world conditions, to evaluate very long term outcomes, and to investigate outcomes that were not the main purpose of the intervention—for example, the impacts on health of changes in education or social welfare policies. These evaluations allow retrospective examination of policies or interventions, and the evaluation of large scale or irreversible interventions such as national policy changes or large public infrastructure investments. They are the approach of choice when a controlled trial is not possible or ethical, or when previous political or financial commitments can make explicit experimentation unattractive to decision makers.

### Concepts and definitions

The new framework uses a broad definition of natural experiments. We use the term to refer to events or processes outside the control of a researcher that divide a population into exposed or unexposed groups, or groups with differing degrees of exposure. A natural experimental evaluation uses data emerging from the introduction, delivery, or withdrawal of a natural experiment to evaluate the impact of the intervention on an outcome or outcomes. Other authors have proposed narrower definitions, such as those based on a list of study designs or on methods that can address unobserved confounding,^29^ or that require intervention assignment to be “as-if randomised.”^30^ We prefer a broader definition for two reasons. Firstly, study design labels do not necessarily indicate study quality, and lists of methods tend to be arbitrary and rapidly become outdated. Secondly, “as-if randomisation” can be difficult to define precisely.^31^ “As-if randomisation” captures an important feature of natural experiments, but represents one end of a spectrum of possible natural experiments rather than characterising the whole range of opportunities that can usefully be exploited with the right choice of methods.^24^ The focus on specific events or processes (known as assignment or allocation processes) that determine exposure distinguishes natural experimental evaluations from the broader range of observational studies.

### Design and planning

**Fig 1:**
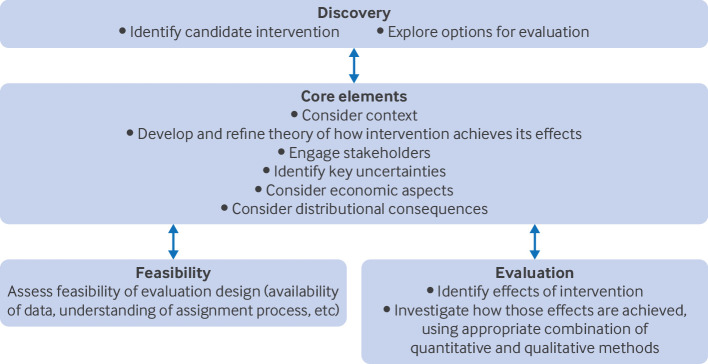
Framework for planning natural experimental evaluations: adaptation of UK Medical Research Council and National Institute for Health and Care Research (MRC/NIHR) framework for developing and evaluating complex interventions^32^

We recommend an approach to planning natural experimental evaluations adapted from the MRC/NIHR framework for the development and evaluation of complex interventions.^32^ That framework highlights the value of a complex systems perspective for evaluation because considering a natural experiment event as a disruption within a system can help identify the breadth of potential intended and unintended impacts, as well as the role of context in shaping the effects of the intervention. Figure 1 presents the stages of identifying and appraising opportunities for a natural experimental evaluation and working out a feasible and appropriate design. Three important phases in the scoping and planning of natural experimental evaluations are identifying and theorising natural experiments, assessing their evaluability, and conducting feasibility studies for a future evaluation.

*Identifying natural experiment*—A variety of circumstances can provide opportunities for a natural experimental evaluation.^24^ We outline five kinds of opportunity that have been widely used. One is where there is a clear division in the presence or type of exposure between otherwise similar subpopulations by time or place of implementation; for instance, when policies such as state level gun control laws are implemented in some areas but not others.^33^ Individual level allocation mechanisms such as eligibility criteria embedded within a policy—for example, means tests that define entitlement to social security benefits^34^ ^35^—provide a second kind of opportunity. A third is the phased implementation of a policy across a population, such as implementation of the UK social security benefit Universal Credit.^36^ ^37^ A fourth is when randomisation is built into the policy, as in the case of a lottery used to allocate housing places.^38^ A fifth is when flaws or shortcomings emerge in policy delivery, such as database errors^39^ ^40^ or false negative test results in the UK’s Test and Trace programme for covid-19.

*Assessing evaluability of natural experiment and feasibility of evaluation*—A formal evaluability assessment is one way of ensuring that natural experimental evaluations are well designed and address relevant questions. Evaluability assessment is a systematic, collaborative approach to evaluation planning that is increasingly used in public health research.^41^ The assessment helps to identify key uncertainties that the evaluation should address, develop a theory of how the intervention works,^32^ and reach consensus about the plausible overall and distributional (ie, equity) effects the intervention could produce, the potential influence of the evaluation on future policy decisions, and how the results might contribute to the wider evidence base.^42^ ^43^ Assessing the evaluability of the natural experiment can help to ensure a shared understanding with stakeholders of what an evaluation can and cannot deliver. The process enables information to be gathered on intervention delivery and the availability of, and access to, monitoring data, and establishes a clear understanding of the assignment process for the intervention. A thorough assessment of the feasibility of the evaluation should be conducted to assess the practicalities of implementing the evaluation design, such as whether routinely collected data adequately capture differences in exposure and outcomes, that the data will enable sufficient statistical power in analyses, and whether alternative methods can be used if the preferred option is not feasible.^43^ As with randomised trials, the funding for natural experimental evaluations might have to incorporate contingency in case the proposed evaluation is unviable; for example, by including an explicit breakpoint in the award when a formal decision of whether to continue would be made.

*Protocols and preregistration*—It is best practice to develop a protocol, or some other form of prior study plan appropriate to the methods being used, and to place it in the public domain before analysis commences. Natural experimental evaluations commonly use several datasets and methods of analysis, and are often retrospective. Publishing analysis plans before data analysis begins enables users to see which analyses reflect previous hypotheses and which have been informed by emerging findings. Protocols can of course be adapted, if need be, provided that amendments are systematically recorded to maintain a transparent record of how the study design has evolved.

*Engaging stakeholders*—In evaluations of natural experiments, there will be a diverse range of stakeholders involved at different stages of the intervention and the evaluation. Relevant stakeholders might include legislators, policy makers, organisations, and individuals responsible for implementation, institutions enabling access to necessary datasets, advocacy groups, representatives of communities affected by the intervention, and the evaluation funder.^44^ Involvement of such stakeholders throughout the evaluation maximises the likelihood of findings being relevant, understood, taken up, and used for decision making. To avoid conflicts of interest, clear boundaries should be agreed for stakeholder involvement and the protocol or evaluation plan made publicly available.^45^

*Taking complex systems perspective*—Population health and health system interventions typically have several components and their impacts are moderated by interactions with elements of the wider system in which they are implemented. When evaluating a natural experiment, considering such interactions can help researchers understand why the intervention succeeds or fails to achieve its intended impact,^46^ or why impacts vary from one setting to another.^47^ For example, if we want to evaluate the introduction of a tobacco tax, we might consider how smokers, retailers, producers, smugglers, the mass media, think tanks, tobacco control advocates, and the cigarette taxation system might react to the introduction of the tax in ways that could dampen or amplify its effects. Taking a systems perspective involves including processes in the evaluation to build an understanding of how the intervention interacts with its context to produce impact. This can include using system mapping^48^ ^49^ (eg, developing a causal loop diagram) to create a robust theory of change for the intervention, identifying outcomes to measure (intended and unintended), or using a system dynamics model to simulate the evolution of the system over time.^50^

### Methods for natural experimental evaluations

Evaluation designs that use both qualitative and quantitative methods are needed to provide an understanding of how the intervention effects were achieved given interactions between the intervention and elements of the wider system. Use of mixed methods can strengthen the estimation of effect sizes by providing a detailed understanding of the assignment process and how far it can be expected to generate otherwise comparable groups of exposed and unexposed units. Single or multiple qualitative and quantitative methods can be combined within an evaluation in several ways, including sequential exploratory, sequential explanatory, parallel convergent (triangulation), and integrated approaches.^51^ ^52^ The value of a mixed methods approach is greater if planned in advance,^53^ for instance, by including an integration work package in the project plan and explicitly earmarking resources for it.^54^ For example, in research to increase understanding of sugar-sweetened beverage taxation policies, simultaneous consideration of several types of data could be used to investigate whether the tax reduces the number of sugar-sweetened beverage consumers, and whether this reduction leads to an increase in the political acceptability of further taxes on sugar-sweetened beverages.^55^

### Quantitative methods

The research question and the nature of the assignment process will determine what methods can be used to obtain quantitative effect estimates in a natural experimental evaluation.^56^ A useful planning tool is the target trial framework, which matches elements of study design to the components of a hypothetical randomised trial.^57^ A variety of study designs and quantitative analytical methods are available. Each method has strengths and limitations, and will be more or less applicable in specific circumstances. An overview is provided in table 1. When selecting methods, it is therefore best to avoid thinking in terms of a hierarchy. Instead, the choice will be determined by the research questions, the circumstances of the evaluation, and the availability of data. Often, the ideal data for a natural experimental evaluation will not be available. Because the assignment processes used in natural experimental evaluations are rarely random, threats to internal validity caused by selective exposure to the intervention are always a concern. Use of a combination of methods (each with differing strengths and limitations) might help address the threats. When reporting the analysis, it is important to state the treatment effect being evaluated by specifying the causal contrast or estimand(s);^56^ and to explain for users whether the main interest is in the average effect of the intervention on an individual or the average effect of the intervention on the population.^58^

### Economic evaluation

Economic evaluations should ideally be conducted in conjunction with evaluations of effectiveness of the natural experiment because there are resource constraints on the implementation of policies. Designing, conducting, and reporting economic evaluations of natural experiments generate specific challenges. Because natural experimental evaluations often assess effects that are by-products rather than directly intended outcomes of the intervention, economic evaluation will often require a broad, “societal,” perspective rather than a sector specific perspective. For example, in an evaluation of the health impacts of Universal Credit, a UK social security programme intended to prevent poverty and provide incentives to work, the economic evaluation examines health and wellbeing, as well as income, employment, and economic productivity outcomes.^59^ Such a broad perspective will require data on multisectoral costs and outcomes, which could be hard to obtain. Routinely collected data can enable a long time horizon over which outcomes are calculated. If suitable data are available, methods such as distributional cost effectiveness analysis^60^ and extended cost effectiveness analysis can be used to investigate the equity impact of an intervention. Challenges involved in designing and conducting an economic evaluation alongside natural experimental evaluation are outlined by Deidda and colleagues,^61^ and guidance has recently been developed for identifying appropriate use and application of complex system models in economic evaluations.^62^

### Qualitative methods

Qualitative methods can strengthen natural experimental evaluations in several ways. They can help researchers to characterise the intervention, understand assignment processes, explore mechanisms, identify threats to validity, help define parameters, and interpret and strengthen causal claims. Evaluations should therefore be planned and conducted in an integrated way to ensure that the qualitative components are incorporated throughout to achieve maximum use of the qualitative data. Below we highlight the components of an evaluation to which qualitative methods contribute beyond their use in process evaluations.^63^ Table 2 provides examples of using qualitative methods. Note that the framework focuses on the use of qualitative methods within evaluations whose primary aim is to estimate effect sizes of interventions, rather than studies whose main goal is to address questions such as the contexts in which interventions work.^47^ ^64^

A key use of qualitative methods is to characterise the intervention in order to understand its rationale and the organisational, historical, political, and social and policy context, including co-occurring interventions, in which it is implemented.^65^ ^66^ Qualitative system mapping techniques (eg, group model building^67^) can be used to identify the elements of contexts that are important to include in the evaluation^68^ and can help to identify important preconditions, assumptions, and potential mechanisms of effect, given the structure of the system.^69^ Qualitative methods can also inform the selection of exposed and unexposed populations, and choice of appropriate outcomes and indicators.^70^ This helps ensure that the chosen quantitative indicators capture what is intended, such as outcomes that are important for stakeholders, and that their strengths and limitations are well understood. In some natural experimental evaluations, qualitative methods are used to generate data on outcomes, to enable triangulation, or to identify secondary outcomes not captured in quantitative datasets or unanticipated at the outset.^71^ Qualitative data might also serve as primary evidence on changes in knowledge, understanding, or practices that are associated with an intervention when quantitative data on such outcomes are not available (table 2).

Most importantly, qualitative methods can help us to understand mechanisms and mediators, and help explain change. Analysis of qualitative data can explore why the intervention did, or did not, lead to anticipated outcomes through making inferences about causal processes from comparisons within the case,^72^ drawing on approaches such as process tracing^73^ or analytic induction.^74^ Appropriate qualitative analysis, in the light of theories of change, strengthens causal inferences and claims about transferability.

### Reporting, critical appraisal, and evidence synthesis

*Reporting*—Accurate, clear, and comprehensive reporting of the natural experiment and the evaluation is crucial for the best use and understanding of the evaluation. Details of reporting guidance likely to be useful to researchers conducting natural experimental evaluations are provided in supplementary file 2.

*Critical appraisal*—A systematic assessment of the design, conduct, and analysis of a study might be required to understand the rigour of an individual study or undertaken as part of evidence synthesis. No single critical appraisal tool can fully assess the risk of bias of all natural experimental evaluation study designs.^75^ ^76^ ^77^ ^78^ For some types of natural experimental evaluations, and some systematic reviews, an appraisal framework more like those used for qualitative research might be more appropriate.^79^ ^80^

*Evidence synthesis*—Synthesising evidence from natural experimental evaluations requires consideration of how to manage the expected diversity in study design and characteristics. For some review topics, it might be more valuable to examine whether there is any evidence for an effect or to explore intervention mechanisms, for example, rather than to estimate an overall effect size within a meta-analysis. Approaches for synthesis without meta-analysis will often be useful; for example, guidance provided by Cochrane^81^ ^82^ and the RAMESES (Realist and Meta-narrative Evidence Syntheses: Evolving Standards) group.^83^ The specifics of the evidence synthesis questions and studies involved will determine the appropriate methods to use, with a mixed methods design often useful.^84^ ^85^

*Certainty of evidence*—If the aim of evidence synthesis is to estimate an effect size, then it is usually appropriate to summarise confidence in the overall effect estimate; that is, to formally assess the overall certainty of the findings. The framework most often used is GRADE (Grading of Recommendations, Assessment, Development, and Evaluations).^86^ Challenges in using GRADE with natural experimental evaluations—including the difficulty of classifying risk of bias for study designs not typically used in epidemiology, selecting outcomes for synthesis, and the lack of differentiation in certainty assessments for population health and health system interventions—are being addressed by the GRADE Public Health Group by developing further guidance and provision of training.^87^

### Data infrastructure and information governance

Natural experimental evaluations often use data collected for other purposes. Such sources include administrative or commercial datasets, population surveys, and data collected from point of sale, traffic sensors, fitness apps, and so on. Secondary analysis of such datasets enables interventions to be evaluated retrospectively using data whose unit cost is a small fraction of the cost of collecting primary data. However, negotiating access to such datasets can often be a time consuming, expensive, and uncertain process, especially if the research involves combining data from several sources. An alternative is to use secure research platforms known as Trusted Research Environments, which are designed to curate data securely and to provide researchers with efficient access. For example, the Brazilian Centre for Data and Knowledge Integration for Health (CIDACS) stores, processes, and links identified data and has secure procedures to provide access to deidentified or anonymised data linking social benefit programmes with deaths, births, and infectious diseases (https://cidacs.bahia.fiocruz.br/en/).

### Good practice considerations

Good practice considerations, derived from the content of the updated framework, are provided for different users. Box 1 presents a condensed form of these recommendations. The main messages for planning, commissioning, conducting, reporting, and using evidence from natural experimental evaluations have been grouped according to the key audience, concentrating on messages that are practical and implementable.

Box 1 **Good practice considerations**

All producers and users of natural experimental evaluations should:

- Understand the design and planning processes of an evaluation of a natural experiment, including how to identify opportunities for natural experimental evaluation, select the most appropriate evaluation approach, and assess the feasibility of the evaluation.
- Consider the needs and perspectives of the full range of stakeholders in the intervention and its evaluation.
- Recognise the respective strengths of quantitative, qualitative, and integrated analytical approaches, incorporating perspectives from diverse disciplines, such as economics, social sciences, and epidemiology, for investigating the impacts of natural experiments.

Researchers conducting natural experimental evaluations should:

- Be aware of the circumstances that are likely to give rise to good opportunities for a natural experimental approach. Adopt methods that are appropriate to the data available and to the processes that determine exposure to the intervention of interest.
- Consider adopting a systems approach to evaluating natural experiments.
- Consider using a combination of methods, including alternative methods of effect estimation, robustness checking, and a mixture of qualitative and quantitative methods.
- Adopt open science practices, publishing a protocol or plan of the study in advance in open access journals or repositories.
- Clearly report the natural experiment event and all stages of the evaluation, including its planning, protocol, analyses, and results, using established reporting standards if available, ensuring key details are in plain language appropriate for the evidence users.
- Include a health equity perspective in the evaluation. Be aware that evaluation of the strength of evidence from natural experimental evaluations should be based on detailed appraisal of the strengths and limitations of the study methods for the specific evaluation, not on generic hierarchies of study design.

Research funders and commissioners supporting and investing in natural experimental evaluations should:

- Encourage best practice when commissioning or funding natural experimental evaluations, for example, by requiring that a protocol or study plan is available before starting the analysis, that findings and analytic scripts are published in open access journals or other suitable platforms, and that the relevant reporting guidelines are followed.
- Establish processes within funding bodies to facilitate flexible and timely responses to prospective natural experimental evaluation opportunities.
- Support capacity building for natural experiments by investing in infrastructure and the workforce.
- Negotiate with data owners to make routinely collected data available and linkable to other datasets for evaluations of policies and programmes.
- When commissioning natural experimental evaluations, be prepared to be flexible and pragmatic, and accept that both the evaluability of the natural experiment and the feasibility of the evaluation require assessment.
- Flexibility might also be required when considering the start date and timescale of the research because policy interventions can be delayed, changed, or withdrawn, and the effects of each will require consideration in an evaluation.

Journal editors, policy makers, practitioners, and other decision makers publishing and using evidence from natural experimental evaluations should:

- Provide guidance for authors and reviewers on requirements for reports of natural experimental evaluations.
- Use evidence from high quality natural experimental evaluations when this is the most appropriate or available form of evidence, being aware of any limitations of the evaluation.
- Incorporate evaluation plans into the implementation of new policies and programmes.

## Conclusion

We have provided an overarching framework for evaluating population health and health system interventions as natural experiments, within which more detailed guidance on specific methods and techniques can be situated. The new framework is aligned with the updated MRC/NIHR framework for evaluating complex interventions in emphasising the value of understanding interventions as events in complex systems, and adopting a plurality of methods with the aim of providing evidence that is useful for decision making. To develop the framework, we consulted widely with producers and users of evidence whose feedback confirmed the usefulness of adopting a broad definition of a natural experiment, rather than limiting the approach to a narrow range of circumstances and techniques. Interest in natural experimental approaches has expanded markedly since the first edition of the guidance was published in 2012. With further investment in research capacity and infrastructure, continued methodological innovation, and a growing appreciation of the range of opportunities presented by service and policy developments, we believe there is scope for considerable further expansion.

## Web extra

Extra material supplied by authors

Web appendix 1: Supplementary file 1: development of framework

Web appendix 2: Supplementary file 2: additional information

## Notes

Contributors: All authors made a substantial contribution to all stages of the development of the framework, contributing to development, drafting, and final approval of the framework and this paper. PC led the conceptualisation of the project, led on concepts and definitions, design and planning, infrastructure, data governance, and led the collation and editing of the framework. MC drafted evidence synthesis and good practice considerations, developed material for and helped facilitate the expert workshops, organised the online consultation, contributed to the content, collation, and editing of the framework, and wrote the first draft of this paper. MD led on economic evaluation. RD led on study registration and protocols. JG led and drafted qualitative methods. SVK drafted mixed methods, co-led on concepts and definitions, qualitative methods, and evidence synthesis. JL co-led quantitative methods. DO co-led design and planning and evidence synthesis. FdV co-led quantitative methods. MW contributed to the conceptualisation of the project, led on ideas for content on guidance for policy makers and other decision makers, and capacity building for optimal use of natural experimental evaluations. PC chaired the expert workshops, MC, MD, RD, SVK, JL, and DO contributed to the expert workshops. PC is the guarantor of this work. The corresponding author attests that all listed authors meet authorship criteria and that no others meeting the criteria have been omitted.

Patient and public involvement: The project was methodological. Throughout the course of the project there was engagement with appropriate audiences, such as relevant policy makers, practitioners, and local and national government representatives. The workshops and consultation exercise included input from evidence users as well as researchers.

Dissemination to participants and related patient and public communities: The authors plan to continue dissemination of the research through a launch event webinar, presentations at conferences and through social media to stakeholders who generate or use evidence, including policy makers, practitioners, and local and national government representatives. The authors are committed to disseminating the findings in formats accessible to relevant audiences to promote broader engagement in natural experimental evaluations.

Provenance and peer review: Not commissioned; externally peer reviewed.
