# Supplementary material for: Using natural experiments to evaluate population health and health system interventions: new framework for producers and users of evidence
Source: BMJ. 2025 Mar 28;388:e080505. doi: 10.1136/bmj-2024-080505 (PMC11950994; doi:10.1136/bmj-2024-080505)
Supplement: Supplementary file 1 — Web appendix 1: Supplementary file 1: development of framework [file crpe080505.ww1.pdf]

Using natural experiments to evaluate population health and health system interventions. A new framework for producers and users of evidence

Supplementary file 1: Development of the framework (abridged from full report,[1] Chapter 2: Methods for developing the framework)

We formed a project writing group, reporting to a funders' oversight group, and convened a stakeholder advisory group. After developing a first draft of the framework, we undertook a consultation process using online workshops and an online consultation to seek recommendations on what content to include in updated guidance for conducting and using natural experimental evaluations.

The project writing group comprised population health researchers representing a range of disciplines and methods, including epidemiology, statistics, health economics, public health, qualitative and evidence synthesis research. Sections of the framework were developed by subgroups of the writing group, with drafts shared with the full group for comment and additional input. Monthly meetings were held throughout the project to assess timeline appraisal and study goals and objectives. This included regular communication with Social and Public Health Sciences Unit staff who assisted with the practicalities and logistics of the online consultation questionnaire data collection.

An advisory group was convened to represent key stakeholders in natural experimental evaluations, including those with experience of using natural experimental evidence in addition to members with methodological expertise. The group met quarterly over the course of the project, with the remit to provide expert input and discuss progress against key milestones of the project. An oversight group was convened by the funders, comprising representatives of the MRC and NIHR, including the MRC-NIHR Methodology Advisory Group, and the UKRI MRC Population Health, Population Health Sciences, the Public Health Intervention Development funding boards and the NIHR Public Health Research Programme. This group met every two months with members of the project team to provide oversight of the content and progress of the project. Members of the advisory and oversight groups are listed in Box S1.

Expert workshops

The writing group developed a first draft of the framework, creating an outline of proposed sections for the framework and key issues for those sections to include. Online expert workshops were held to obtain expert opinion from international researchers, policymakers, funders, and journal editors,

on the first draft of the framework, a key decision point in the development of the framework. The objectives were to identify additional content, unnecessary content, any topics of contention, and any required revisions to the structure of the framework.

There were three workshops, each with the same content, to enable participants from different world time zones (North and South America, Europe and Africa, and Australasia) to take part at a suitable time. In advance of each workshop, participants were provided with a briefing document summarising the project objective, with questions for the workshop sessions based on the above objective plus specific issues for the proposed framework chapters and a copy of an article by the project team highlighting issues relating to natural experimental evaluations.[1] The questions used to prompt discussion are presented in Table S1.

The briefing document and the workshop format were split into three sessions:

- Session A examined coverage of the framework and concepts and definitions used in the framework guidance.
- Session B focussed on processes relating to natural experimental evaluations: design and planning, pre-registration of evaluations, infrastructure and information governance for the use of administrative data, and engaging policymakers and other stakeholders.
- Session C focussed on methods: quantitative, qualitative, mixed methods, economic evaluations, reporting and evidence synthesis.

Respondents were recruited for the workshop through co-investigator and advisory group networks and individuals identified as the framework was developed. Authors of academic articles reporting natural experimental evaluations in population health were identified as the framework content was developed and invited to participate in the workshop. Relevant stakeholders in the research community were also recruited, representing funders from international funding boards, journal editors and peer reviewers of journal reporting on natural experimental evaluations. Knowledge within the project team and project advisory group was used to add to the list of people with professional expertise of natural experimental evaluations population health interventions. A total of 72 individuals were invited to attend, 23 accepted the invitation and 21 participants attended. Each workshop lasted approximately three hours.

Table S1: Workshop questions

**Session A Coverage, concepts and definitions**
Coverage
1. Does the proposed list of sections provide a comprehensive and coherent structure for the guidance?

2. Are there any key issues that do not fit within the proposed structure?<br>***Concepts and definitions***<br>1. Is this broad definition of natural experimental evaluations appropriate? If not, how can it usefully be made more specific?<br>2. Would it be useful to include in the guidance a glossary along the lines of Table 3? If so, are there any key terms missing? Are brief summaries of the kind illustrated likely to be useful. Would links to references for more formal definitions be helpful?
**Session B: Process**<br>***Design and planning***<br>1. Does the framework provide a useful structure for planning and conducting a natural experimental evaluation? Are there other aspects of design and planning natural experimental evaluations that should be addressed in this guidance?<br>2. Have we identified the most important sets of circumstances that provide opportunities for natural experimental evaluations? Are there others we should add to the list?<br>***Pre-registration of natural experimental evaluations***<br>1. How can trial registration systems, designed for prospective studies, be adapted to the requirements of retrospective studies?<br>2. How can the benefits of registration be aligned with need for flexibility in study protocols?<br>3. What additional safeguards, if any, are needed to ensure transparency in the conduct and reporting of natural experimental evaluations?<br>4. What can funders and journal editors do to support transparency in the conduct and reporting of natural experimental evaluations?<br>Infrastructure and information governance for the use of administrative data<br>1. What are the most important barriers to the efficient use of routinely collected data for natural experimental evaluations?<br>2. How can these barriers be removed or minimised, while still preserving data security?<br>3. Are there examples of good practice (e.g., effective infrastructures for making data available, or streamlined approaches to information governance) that we should highlight in the guidance?<br>***Engaging policymakers and other stakeholders***<br>1. Are there good examples of guidance on the use of natural experimental evaluations to inform policy making from countries other than the UK?<br>2. What other approaches might help to improve the understanding and use of natural experimental evaluation evidence by decision-makers, especially those outside central government?
**Session C: Methods**<br>***Quantitative methods***<br>1. Are any important methods missing from the list above? Have we included any that do not belong (e.g., because they could not be used to identify a causal effect in any circumstances)?

2. Is there a hierarchy among the methods, or should they be seen as a toolkit?

3. Is a target trial framework as useful way of evaluating natural experimental evaluations study designs? Are there more straightforward alternatives?

Economic evaluation of natural experimental evaluations

1. The MRC/NIHR framework for developing and evaluating complex interventions recommends CCA and CBA as the most appropriate forms of economic evaluation, and the adoption of a broad (e.g. societal) rather than a narrow (e.g. provider) perspective for identifying costs and outcomes. Do these recommendations also apply to natural experimental evaluations?

2. Does the incorporation of economic evaluation into a natural experimental evaluation pose specific challenges over and above those associated with any evaluation of a complex Intervention. Are there good examples of how these have been addressed?

Qualitative methods

1. The guidance currently focuses on practical issues and not on methodological theory around qualitative natural experimental evaluation design. Do you agree with this emphasis?

2. The section is aiming to be 'theory neutral' and does not compare different theoretical frameworks or designs, e.g., realist designs, normalization process theory, etc. Do you agree?

3. Can a natural experimental evaluation rely entirely on qualitative methods, for example to understand change, mechanisms and mediators, or the perspectives and practices of intervention stakeholders, or should such methods always be used in conjunction with quantitative methods of effect estimation?

Integrated, mixed- and multi-method evaluations

1. Practicalities aside, are any of the methods listed clearly preferable to the others?

2. Taking practicalities into account, which method or methods are most appropriate to natural experimental evaluations?

Reporting

1. Are existing reporting guidelines adequate for reporting natural experimental evaluations, or is there value in extending them to cover issues specific to natural experimental evaluations?

Critical appraisal and evidence synthesis

1. What balance would users find most useful between conceptual and practical issues in guidance for appraising and synthesising natural experimental evaluation evidence?

2. To what extent are the challenges for critical appraisal and evidence synthesis of natural experimental evaluations particular to NEEs?

3. Is it best to use ROBINS-I and GRADE to assess risk of bias and certainty of evidence, despite the challenges involved, or are there other approaches that are sufficiently rigorous but more straightforward?

Online consultation

Following revisions to the framework in response to feedback from the workshops, an online consultation was developed to consolidate the content of the framework. A draft of the framework was provided in which each section had a bullet point introduction of key messages, giving the option to continue reading the full text of the section, answer questions and provide comments on the section, or move forward to the next section. Questions asked of the participants are provided in Table S2. The respondents were invited to provide their details to be included in acknowledgements (see Table S3), or alternatively to complete the consultation anonymously. All responses were de-identified and no statements were linked to particular respondents.

The consultation was open for seven weeks, from 21st September 2022 until 8th November 2022. An initial list of 95 individuals were invited to take part, including individuals involved in commissioning, conducting, and using evidence from natural experimental evaluations, i.e., researchers, local and national government representatives and practitioners, policymakers, journal editors and funder representatives. Invitees were identified from among those invited to the previous workshops, recommendations from the project advisory group, and 'snowball' invitation suggestions from participants. Members of the NIHR School for Public Health Research (SPHR) Network for the use of Natural Experiments in Public Health were also invited. By the closing date 200 people had been invited to participate; the intention was to share the consultation widely and a high response rate was not anticipated.

Participants were invited by email, outlining the purpose of the consultation and inviting them to reply indicating whether they would take part. When the participant accepted, their email address was added to an access list for the consultation and they were provided a link to the online consultation questionnaire. The introductory page of the questionnaire provided a clear plain language statement explaining the consultation and a pdf of full participant information details relating to the consultation.

There were 44 completed responses. Participants provided details of their roles; many provided information about multiple roles. Most respondents were researchers (intervention n=18, quantitative n=39, qualitative n=17), and several were members of funding boards (n=6), representatives of a funding body (n=1), journal editors (n=8), policymakers (n=1), practitioners (n=2), or clinicians (n=5). Most respondents reported a university as their institution (n=41) although

some participants had dual or multiple roles, and some respondents reported their organisations as public sector (n=5), not for profit (n=2), or a for-profit organisation (n=2).

Finalising the framework

De-identified feedback from the online consultation was summarised and collated to gather advice on the content of the framework. The writing team used the feedback from the consultation to revise the guidance accordingly. Throughout the project stages, drafts of the framework were provided to the advisory group and the oversight group for feedback.

The good practice considerations were developed by the writing team as summaries of key messages contained in the framework for different users of the framework. The good practice considerations were included in the online consultation to give participants the opportunity to comment and make further suggestions.

References

1. Craig P, Campbell M, Deidda M, et al. Using natural experiments to evaluate population health interventions: a framework for producers and users of evidence. Public Health Res 2025 (in press)
2. Craig P, Campbell M, Bauman A, Deidda M, Dundas R, Fitzgerald N, et al. Making better use of natural experimental evaluation in population health. BMJ 2022;379:e070872.

Box S1: Advisory group and oversight group members

Advisory group members:
Adrian Bauman, University of Sydney
Kate Tilling, University of Bristol
Marc Suhrcke, Luxembourg Institute of Social and Economic Research
Niamh Fitzgerald, University of Stirling
Sara Shaw, University of Oxford
Audrey Ceschia, Editor Lancet Public Health
Scott Lloyd, Middlesbrough Council and Associate Lead for Public Health Research, NIHR Clinical Research Network North East & North Cumbria
Sarah Sharples, CSA Dept for Transport

Oversight group members:
Graham Hart (Chair), MRC PHIND panel
Peymané Adab, MRC-NIHR Methodology Advisory Group
Claire Kidgell, Assistant Director NIHR
Catherine Moody, Head of Population Health UKRI MRC
Tamsyn Derrick, Programme Manager Population Health Sciences and PHIND UKRI MRC
Peter Craig, representing project team, MRC/CSO SPHSU, University of Glasgow

Table S2: Consultation questions

Each of the 8 framework sections were accompanies by the same questions asking:<br><br>Do you agree with the content of the proposed section?<br>• Agree<br>• Agree, but some additional content or explanation could be provided (please explain)<br>• Disagree (please explain)<br>• Don't know<br><br>Please explain any agreement, disagreement, or additional comments you have about the content of this section	
Additional questions:	
Consultation section: Concepts and definitions	If there are any key terms missing from the glossary, please describe in the comments section below.
Consultation section: Qualitative methods	Are there good examples for the use of qualitative methods within NEEs in Low/Middle Income countries which you would recommend? Please provide details in the comments section below.<br><br>Are there good examples of the use of qualitative methods within NEEs in health services/systems research which you would recommend? Please provide details in the comments section below.
Consultation section: Good practice considerations	Please suggest amendments and/or further recommendations that you think we should emphasise, based on the content of the earlier sections.

Table S3: Consultation participants' roles and organisations

**Participant role (some provided multiple responses)**	
**Role**	**No.**
Member of funding board	6
Funding body representative	1
Journal editor	8
Intervention researcher	18
Quantitative researcher	39
Qualitative researcher	17
Policymaker	1
Practitioner	2
Clinician	4

Other	1
Details provided for ‘Other’	Public Health Consultant
**Participant organisation (some provided multiple responses)**	
**Organisation**	**No.**
University	41
Public sector organisation	5
Non-profit organisation	2
For-profit	2
Other	0
