# Supplementary material for: Using natural experiments to evaluate population health and health system interventions: new framework for producers and users of evidence
Source: BMJ. 2025 Mar 28;388:e080505. doi: 10.1136/bmj-2024-080505 (PMC11950994; doi:10.1136/bmj-2024-080505)
Supplement: Supplementary file 2 — Web appendix 2: Supplementary file 2: additional information [file crpe080505.ww2.pdf]

Using natural experiments to evaluate population health and health system interventions. A new framework for producers and users of evidence

Supplementary file 2

Table S4: Reporting guidance likely to be useful to researchers conducting natural experimental evaluations

**Reporting guidance**	**Focus**
SPIRIT 2013: Standard Protocol Items: Recommendations for Interventional Trials[1]	This guidance for reporting clinical trial protocols may be useful for identifying key features of the natural experimental evaluation to include in a protocol. A reporting guideline is in development for protocols for observational studies (SPIROS)[2]
STROBE: Strengthening the Reporting of Observational Studies in Epidemiology[3]	A list of 22 items to aid the reporting of observational studies - cohort, case–control, or cross-sectional study design.
TREND: Transparent Reporting of Evaluations with Nonrandomized Designs[4]	Checklist for reporting non-randomised behavioural and public health intervention evaluations.
RECORD: REporting of studies Conducted using Observational Routinely-collected health Data[5]	An extension to STROBE, this guidance provides a checklist (13 items) for studies using routinely collected data
TIDieR-PHP: Template for Intervention Description and Replication - population health and policy[6]	An adaptation of the TIDieR guidance,[7] TIDieR-PHP is a reporting guideline for evaluation studies of population health and policy interventions, such as legal, fiscal, structural, organisational, environmental, or policy interventions
CHEERS 2022: Consolidated Health Economic Evaluation Reporting Standards 2022 (CHEERS 2022)[8]	Guidance and 28 item checklist for reporting economic evaluations of health interventions
SRQR: Standards for Reporting Qualitative Research[9]	Guidance for reporting qualitative research that is intended to be relevant across differing paradigms and methods
Triple C (Case study, Context, Complex interventions) reporting principles[10]	Reporting principles for case study evaluations of the role of context in complex interventions
PRISMA-P: Preferred Reporting Items for Systematic reviews and Meta-Analyses for Protocols 2015[11]	Guidance for reporting systematic review protocols
PRISMA 2020[12]	Guideline and checklist to promote complete reporting of systematic reviews
SWiM: Synthesis without Meta-analysis[13]	Guidance for reporting synthesis without meta-analysis in systematic reviews

Supplementary file references

1. Chan A-W, Tetzlaff JM, Gøtzsche PC, Altman DG, Mann H, Berlin JA*, et al.* SPIRIT 2013 explanation and elaboration: guidance for protocols of clinical trials. *BMJ : British Medical Journal* 2013;**346**:e7586. https://doi.org/10.1136/bmj.e7586
2. Mahajan R, Burza S, Bouter LM, Sijtsma K, Knottnerus A, Kleijnen J*, et al.* Standardized protocol items recommendations for observational studies (SPIROS) for observational study protocol reporting guidelines: protocol for a Delphi study. *JMIR Research Protocols* 2020;**9**:e17864.
3. Elm Ev, Altman DG, Egger M, Pocock SJ, Gøtzsche PC, Vandenbroucke JP. Strengthening the reporting of observational studies in epidemiology (STROBE) statement: guidelines for reporting observational studies. *BMJ* 2007;**335**:806-8. https://doi.org/10.1136/bmj.39335.541782.AD
4. Des Jarlais DC, Lyles C, Crepaz N, Group T. Improving the reporting quality of nonrandomized evaluations of behavioral and public health interventions: the TREND statement. *American Journal of Public Health* 2004;**94**:361-6.
5. Benchimol EI, Smeeth L, Guttmann A, Harron K, Moher D, Petersen I*, et al.* The REporting of studies Conducted using Observational Routinely-collected health Data (RECORD) statement. *PLoS Medicine* 2015;**12**:e1001885.
6. Campbell M, Katikireddi SV, Hoffmann T, Armstrong R, Waters E, Craig P. TIDieR-PHP: a reporting guideline for population health and policy interventions. *BMJ* 2018;**361**.
7. Hoffmann TC, Glasziou PP, Boutron I, Milne R, Perera R, Moher D*, et al.* Better reporting of interventions: template for intervention description and replication (TIDieR) checklist and guide. *BMJ* 2014;**348**.
8. Husereau D, Drummond M, Augustovski F, de Bekker-Grob E, Briggs AH, Carswell C*, et al.* Consolidated Health Economic Evaluation Reporting Standards 2022 (CHEERS 2022) statement: updated reporting guidance for health economic evaluations. *International Journal of Technology Assessment in Health Care* 2022;**38**.
9. O'Brien BC, Harris IB, Beckman TJ, Reed DA, Cook DA. Standards for reporting qualitative research: a synthesis of recommendations. *Academic Medicine* 2014;**89**:1245-51.
10. Shaw S, Paparini S, Murdoch J, Green J, Greenhalgh T, Hanckel B*, et al.* TRIPLE C Reporting Principles for Case study evaluations of the role of Context in Complex interventions *BMC Medical Research Methodology* 2023;**23**:115.
11. Shamseer L, Moher D, Clarke M, Ghersi D, Liberati A, Petticrew M*, et al.* Preferred reporting items for systematic review and meta-analysis protocols (PRISMA-P) 2015: elaboration and explanation. *BMJ* 2015;**349**.
12. Page MJ, Moher D, Bossuyt PM, Boutron I, Hoffmann TC, Mulrow CD*, et al.* PRISMA 2020 explanation and elaboration: updated guidance and exemplars for reporting systematic reviews. *BMJ* 2021;**372**:n160. https://doi.org/10.1136/bmj.n160
13. Campbell M, McKenzie JE, Sowden A, Katikireddi SV, Brennan SE, Ellis S*, et al.* Synthesis without meta-analysis (SWiM) in systematic reviews: reporting guideline. *BMJ* 2020;**368**.
